# Susceptibility of the Most Popular Soybean Cultivars in South-East Europe to *Macrophomina phaseolina* (Tassi) Goid

**DOI:** 10.3390/plants12132467

**Published:** 2023-06-28

**Authors:** Jovana Šućur Elez, Kristina Petrović, Marina Crnković, Slobodan Krsmanović, Miloš Rajković, Željko Kaitović, Đorđe Malenčić

**Affiliations:** 1 Department of Field and Vegetable Crops, Faculty of Agriculture, University of Novi Sad, 21000 Novi Sad, Serbia; jovana.sucur@polj.uns.ac.rs (J.Š.E.); marina.crnkovic@polj.uns.ac.rs (M.C.); djordje.malencic@polj.uns.ac.rs (Đ.M.); 2 Institute of Field and Vegetable Crops, National Institute of the Republic of Serbia, 21000 Novi Sad, Serbia; slobodan.krsmanovic@agromarket.ba (S.K.); rajkovicmilos@gmail.com (M.R.); 3 Breeding Department, Maize Research Institute, 11185 Belgrade, Serbia; zkaitovic@mrizp.rs; 4 BioSense Institute, University of Novi Sad, 21101 Novi Sad, Serbia; 5 Sector for Plant Nutrition, Agromarket BiH, 76300 Bijeljina, Bosnia and Herzegovina; 6 Department for Research and Development in Agriculture, Institute of Medicinal Plant Research, 11000 Belgrade, Serbia

**Keywords:** soybean tolerance, lipid peroxidation (LP), oxidative stress, superoxide dismutase (SOD), superoxide anion radical (O_2_^•−^)

## Abstract

Oxidative stress in soybean seedlings and the length of the soybean stem lesions infected with the fungus *Macrophomina phaseolina* (Tassi) Goid were evaluated to determine the most tolerant soybean cultivar to this pathogen. The level of superoxide anion radical (O_2_^•−^) production, the activity of the antioxidant enzyme superoxide-dismutase (SOD), and the intensity of lipid peroxidation (LP) were measured in four soybean cultivars: Favorit, Atlas, Victoria, and Rubin. Results showed that O_2_^•−^ radical production and SOD activity were the most elevated in the cv. Favorit inoculated with *M. phaseolina*, while the level of lipid peroxidation intensity was the lowest compared to the control. This indicates that the soybean cv. Favorit has managed to prevent infection with *M. phaseolina*. Furthermore, higher O_2_^•−^ radical production and lower SOD enzyme activity were measured in cv. Victoria, with enhanced lipid peroxidation. This means that the cv. Victoria was infected with *M. phaseolina*, and was the most sensitive. None of the tested oxidative stress parameters showed a significant difference in the cvs. Atlas and Rubin compared to the control. Furthermore, the highest lesion length was measured in the cv. Victoria, followed by cv. Favorit, while the lowest lesion length was measured in the cv. Atlas followed by the cv. Rubin; and thus, the cv. Atlas followed by the cv. Rubin, were the most tolerant soybean cultivars to this pathogen.

## 1. Introduction

**Figure 1:**
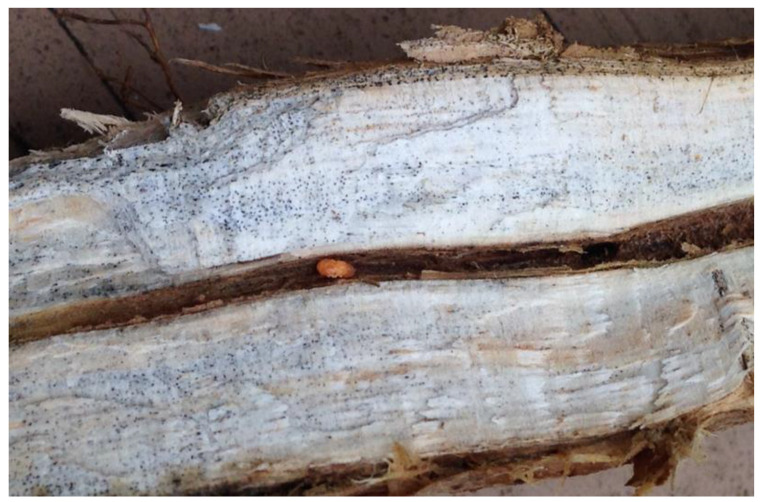
Grey discoloration and black microsclerotia of *M. phaseolina* on the cross-section of the lower portion of a soybean stem.

Soybean (*Glycine max* (L.) Merrill) is one of the oil-seed crops that is cultivated around the globe. Besides having a high content of oil (18–22%), soybean seed contains approximately 40–45% of proteins, which is why this plant is also regarded as a protein crop and is produced in large quantities [1]. The main soybean growing areas are in the U.S., Argentina, Brazil, China, and India [2], while Serbia was the 15th largest producer with 719,370 metric tons in 2021 (https://www.reportlinker.com/clp/country/3685/726451 (accessed on 12 June 2023). However, persistent drought from May to August 2022 has damaged most of the spring crops in Serbia, primarily maize, soybean, and fruits (https://www.fas.usda.gov/data/serbia-serbia-grain-summer-update-2022 (accessed on 12 June 2023). Drought is not only an abiotic factor that can independently reduce soybean yield, but is a predisposing factor that stimulates the infection of the fungus *Macrophomina phaseolina* (Tassi) Goid. This pathogen is one of the devastating soil-borne diseases of soybean, as well as over 500 other plant species, causing charcoal rot, dry root rot, dry weather wilt, ashy stem blight, and seedling blight, whose severity and incidence has increased recently due to climate change [3]. Infected plants have a reduced leaf and seed size and may wilt and prematurely die [4]. Aboveground symptoms of charcoal rot appear after flowering (soybean growth stage R1) and are particularly evident in soybean fields at the R5 (beginning seed), R6 (full seed), and R7 (beginning maturity) growth stages. It is primarily soil-inhabiting polyphagous fungus, but also a seed-borne organism that produces microsclerotia in root and stem tissues [1]. Microsclerotia are black spore-like mycelial structures that allow the fungus to overwinter in the soil [5]. It is a major source of inoculum of *M. phaseolina* and can remain viable in the soil for up to four years after the harvest (Figure 1).

*Macrophomina phaseolina*, the causal agent of charcoal rot on soybean, is an economically important pathogen in Serbia causing the decrease of yields up to 30–50%. It is more prevalent in heat-stressed and drought-stressed conditions. It has been reported that drought-tolerant soybean genotypes are more resistant to this disease than drought-sensitive ones, but no significant differences exist between them [6]. Moreover, *M. phaseolina* is not only a serious threat to soybean, but also to maize, sunflower, and sugar beet [7]. It is a necrotrophic fungal pathogen that takes nutrients from a dead tissue of a host plant, which means that programmed cell death (PCD) induced by plant immunity does not help defense against this kind of pathogen. Since it is more difficult to protect a plant from necrotrophic pathogens, significant losses have been inflicted, not only on soybeans, but on many other widespread crops, such as wheat and barley. Therefore, this pathogen has the greatest economic impact on agriculture [3,8]. In Serbia, it was estimated that crop yields (maize, soybean, and fruits) declined by 20–30%, with total losses reaching USD billions in 2022 (https://www.fas.usda.gov/data/serbia-serbia-grain-summer-update-2022 (accessed on 12 June 2023).

Strategies for charcoal rot management, such as biological control and fungicide application, have all shown limited success [9]. Because of that, growing soybean genotypes tolerant to charcoal rot is the most sustainable strategy in terms of the protection of high yields and seed quality. However, there are no resistant soybean cultivars available in the market [9].

The mechanism used by *M. phaseolina* to infect plants is still unknown, but it has been reported that this fungus produces certain mycotoxins that may play a role in the infection of plants through the roots. Which toxin will be produced depends on the origin of the isolate of *M. phaseolina*, since every isolate produces different toxins. For instance, the isolates recovered from soybean produced mellein, (−)-botryodiplodin, kojic acid, moniliformin, and other secondary metabolites [10,11], while phaseolinone was not detected [5].

The first response of a plant attacked by a pathogen is the induction of an oxidative burst, which ends with cell death at the attack site. The whole process begins with incompatible interaction between plant receptor and toxin from a pathogen, whereby a plant recognizes an attacker. Afterward, there is an increased production of reactive oxygen species (ROS), such as superoxide anion radical (O_2_^•−^) and hydrogen peroxide (H_2_O_2_), with the aim of preventing the spreading of the fungus in plant tissue. It activates the antioxidant defense system of the plant to neutralize the damage caused by ROS. Antioxidants could be enzymes such as superoxide-dismutase (SOD), catalase (CAT), and various peroxidases (PX). On the other hand, antioxidants could be non-enzymatic molecules, such as glutathione, tocopherols, carotenoids, ascorbate, and phenolic compounds [7]. With failure to prevent further spreading of the fungus, as well as the oxidative burst at the infected site, plant tissue suffers the peroxidation of membrane lipids and cell death. This consequently triggers further spreading of the fungus.

This research aimed to compare the sensitivity of four popular commercial soybean cultivars in southeast Europe from different maturity groups (MG) (Favorit MG ’000’, Atlas MG ’0’, Victoria MG ’I’, and Rubin MG ’II’) to the fungus *M. phaseolina*, in order to select the most tolerant cultivar to this pathogen and the most suitable for production, as far as this pathogen is concerned. This was done by comparing two different parameters: (1) the stem lesions obtained in standard cut-stem inoculation method in the V2 growth stage of soybean, and (2) the biochemical response in soybean seedlings after seed inoculation, where three oxidative stress parameters were measured (level of O_2_^•−^ radical production, the SOD activity, and lipid peroxidation).

## 2. Results

**Figure 2:**
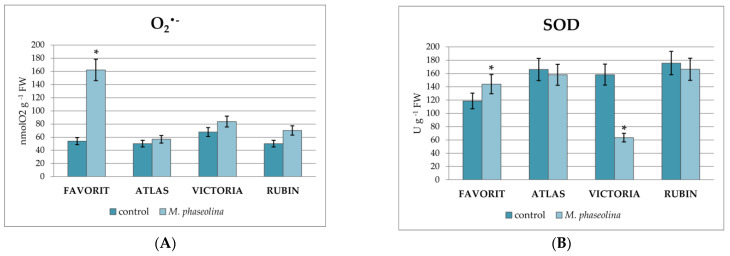

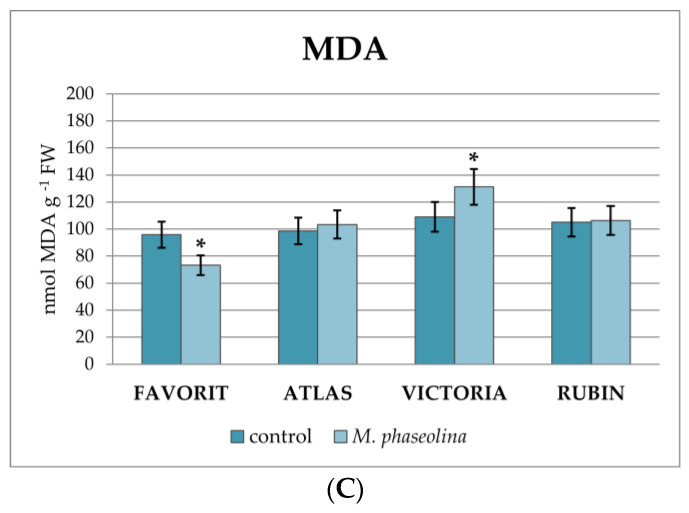
Effect of inoculation of soybean seedlings with *M. phaseolina* on superoxide radical production (nmol O_2_^•−^ g^−1^ FW) (**A**), superoxide-dismutase activity (SOD) (U g^−1^ FW) (**B**), and lipid peroxidation (nmol MDA g^−1^ FW) (**C**). Values mean ± SD of three replicate assays. Labeled columns are significantly different from control (*) (Duncan test, *p* < 0.05).

Visual assessment of the infected soybean seeds showed no differences compared to the control (non-infected seeds). Seed rot and seedling necrosis were not detected. However, the highest production of the superoxide anion radical (162.26 ± 14.72 nmol O_2_^•−^ g^−1^ FW) compared to the control (53.99 ± 3.96 nmol O_2_^•−^ g^−1^ FW) and the highest increase in SOD activity (143.94 ± 5.93 U g^−1^ FW) compared to control (118.52 ± 8.19 U g^−1^ FW) was measured in the cv. Favorit (Figure 2A,B). In the cvs. Atlas, Victoria, and Rubin there were no significant differences in the superoxide anion radical production compared to the control seedlings.

The activity of SOD in the cvs. Atlas and Rubin seedlings did not differ significantly from their controls. Moreover, the activity of SOD in the cv. Favorit was higher (143.94 ± 13.3 U g^−1^ FW) compared to the control seedlings (118.52 ± 10.7 U g^−1^ FW), and in the cv. Victoria was significantly lower (63.47 ± 5.23 U g^−1^ FW) compared to the control (158.19 ± 1.47 U g^−1^ FW).

The highest level of malondialdehyde (MDA) (131.14 ± 117.27 nmol MDA g^−1^ FW), as the marker of membrane lipid peroxidation intensity, was measured in the cv. Victoria compared to the control (109.04 ± 9.51 nmol MDA g^−1^ FW) (Figure 2C) and was significantly higher compared to other cultivars. The lowest MDA content was measured in cv. Favorit (73.25 ± 5.93 nmol MDA g^−1^ FW), compared to the control (95.80 ± 8.19 nmol MDA g^−1^ FW) and this is the lowest MDA content compared to all other tested cultivars. There were no significant differences in the MDA content in the cvs. Atlas and Rubin and their controls.

**Figure 3:**
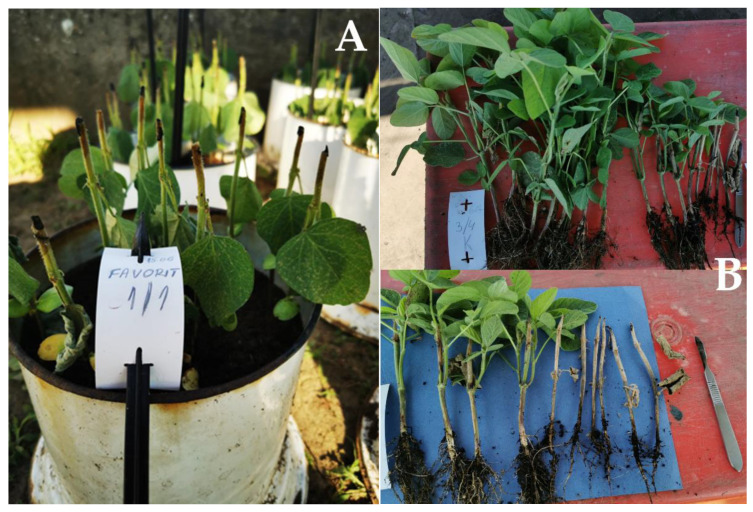
Measurement of lesions caused by *M. phaseolina* on soybean stem: (**A**) lesions after seven days; and (**B**) at the end of the trial (three weeks after inoculation).

Visual monitoring of the disease development on soybean plants and the measurement of stem lesions caused by *M. phaseolina* were presented in Figure 3 and Table 1 (the raw data available at https://doi.org/10.5281/zenodo.5035320 (accessed on 12 June 2023). Statistical data processing led to the conclusion that the most sensitive soybean cvs. were Victoria and Favorit (mean lesion length: 117.7 and 98.8), followed by the cv. Rubin, while the most tolerant was the cv. Atlas. According to the statistical analysis, there are positive correlations between the length of the stem lesions and the activity of SOD, and between the production of the superoxide anion radical and lipid peroxidation process (significant values are marked with red, Table 2). The increase in lesion length correlates with a decrease in SOD activity. Thus, the most sensitive was the cv. Victoria with the longest measured lesion length and the lowest SOD activity.

## 3. Discussion

This study analyzed the tolerance of four commercial soybean cultivars (popular in production in Serbia) to *M. phaseolina*, an important soybean fungal pathogen. In recent years, more frequent droughts have occurred in Serbia. Due to this abiotic stress, soybean plants are more susceptible to infection by *M. phaseolina*, which causes charcoal rot. Symptoms of the disease are usually observed in late-maturing soybean cultivars (MG ‘I’ and ‘II’) towards the end of the growing season. However, this does not mean the early-maturing cultivars (MG ‘000’ and ‘0’) are tolerant. It is considered that they avoid the moment of infection due to the short growing season. For this reason, this study tested the sensitivity of four popular commercial soybean cultivars from different maturity groups (Favorit MG ‘000’, Atlas MG ‘0’, Victoria MG ‘I’, and Rubin MG ‘II’). This approach will allow the comparison of the long-term observations of these four cultivars in the field with trials performed in control conditions and biochemical markers.

The sensitivity of soybean cultivars was screened using the comparation of results of two different inoculation methods: (1) seed inoculation, where three oxidative stress parameters were measured (level of O_2_^•−^ radical production, the SOD activity, and lipid peroxidation), and (2) the standard cut-stem method in the V2 growth stage of soybean, where the stem lesions were obtained.

The visual screening of infected soybean seeds showed no differences compared to the control (non-infected seeds). Seed rot and seedling necrosis were not detected. However, oxidative stress parameters showed that different soybean cultivars had different responses against the fungus pathogen *M. phaseolina*. The highest production of the superoxide anion was in the cv. Favorit. A high level of ROS was expected since oxidative stress is the first response of the plant. Increased production of O_2_^•−^ radicals is usually correlated with an increased activity of SOD. Enzyme SOD catalyzes the removal of O_2_^•−^ radicals, their conversion in a hydrogen–peroxide and a water molecule, thus preventing the formation of a more toxic and the most reactive free radical–hydroxyl radical (^•^OH). Hydroxyl radical causes lipid peroxidation of cellular membranes disrupting membrane integrity. Bearing that in mind, increased SOD activity was expected in the cv. Favorit, since these seedlings had the highest production of O_2_^•−^. In the cvs. Atlas, Victoria, and Rubin there were no statistically significant differences in the superoxide anion production, compared to control seedlings. The activity of SOD in the cvs. Atlas and Rubin did not differ significantly from the control. The activity of SOD was significantly lower in the cv. Victoria, compared to control seedlings, so the enzymatic protection is less compared to other soybean cultivars, resulting in a greater lipid peroxidation and greater stress in plants.

These results agree with previous results of Malenčić et al. [12], who reported that the activity of SOD increased in different soybean cultivars (1511/99, Meli, Alisa, and Sava) infected with the necrotrophic fungus *Sclerotinia sclerotiorum*. Similar results (high SOD activity and a low rate of LP) were obtained earlier [13] for several soybean cultivars (Erie, Holfax, Flint, and Labrador), affected by the abiotic stress (drought). On the other hand, a recent study showed increased enzyme activity levels, which includes SOD, as well as higher content of MDA compared to controls, in four genetically diverse soybean genotypes (PI567731, PI416937, PI567690, and PI408105A) under combined drought and salinity [14]. Similar results were obtained in the study of the reproductive system (anthers and ovules) of cold-stressed chickpea [15]. Furthermore, Helepciuc et al. [16] reported that fungal elicitors in soybean increased the activity of SOD compared to the control, but the activity decreased at higher concentrations of the elicitor used. On the other hand, the same fungal elicitors increased peroxidase activity, the other tested enzyme involved in oxidative stress control, and activity was independent of the concentration of the elicitor used [16].

Malenčić et al. [7] reported that soybean cvs. Meli and Balkan showed tolerance to *M. phaseolina* and the inoculation did not affect the activity of the antioxidant enzyme SOD. Dey et al. [17] reported that a series of different micro RNAs were activated to target the concerned genes which provide defense in jute (*Corchorus capsularis* L.) infected by *M. phaseolina*. Activated micro RNAs initiated multi-layered defense and built strong barriers against *M. phaseolina* mediated by nucleotide binding site (NBS), leucine-rich repeat (LRR) motifs, and the gene regulation of reactive oxygen species (ROS) [18].

One of the main end products of lipid peroxidation process is MDA, whose content is measured to prove level of LP, and its high level is the main indicator of cell damage. Among all investigated soybean cultivars, seedlings of the cv. Victoria had the greatest level of MDA. Thus, the slightly increased production of O_2_^•−^ and decreased SOD activity, compared to control, resulted in a high level of lipid peroxidation process in the cv. Victoria seedlings. On the other hand, the lowest content of MDA was measured in the cv. Favorit seedlings; though these seedlings had the highest production of O_2_^•−^ radicals and higher SOD activity, compared to the control. This is because the O_2_^•−^ radical does not directly cause lipid peroxidation but only leads to the formation of more toxic ^•^OH radical, which causes it. The formation of ^•^OH radical was prevented by the higher activity of the antioxidant enzyme SOD. Among all analyzed soybean cultivars, the cv. Victoria seedlings were the most susceptible to the tested *M. phaseolina* isolates. Moreover, these results indicated that the cv. Favorit was sufficiently capable to defend itself against *M. phaseolina* attack. The lipid peroxidation process in the cvs. Atlas and Rubin did not differ significantly from the control. The different sensitivity of various soybean cultivars to *Agrobacterium tumefaciens* lines was confirmed in the study of Wang et al. [19].

The visual monitoring of the disease development on soybean plants and the measurement of stem lesions caused by *M. phaseolina* led to the conclusion that the most sensitive soybean cvs. are Victoria and Favorit, followed by the cv. Rubin, while the most tolerant was the cv. Atlas. Complete tolerance was not found among tested soybean cultivars and these findings agree with the Twizeyimana et al. [20] study, who reported that tested soybean accessions (Spencer, Croton, Pharoah, DT97-4290, DT98-7553, DT99-17554, DT99-17483, DT99-16864, LS98-3257, LS98-2574,LS98-0719, LS98-1430, LS92-1088, LS94-3207) developed stem necrosis during the experimental time indicating that there was no complete resistance to *M. phaseolina*. Once in the host, *M. phaseolina* releases various toxins and cell wall degrading enzymes disrupting host defense, resulting in cell death [18]. There is a recent reported technique, hyperspectral spectroscopy [21], for the detection of resistant soybean cultivars to fungus *M. phaseolina,* after seedlings’ exposure to its toxins.

It was found that the tillage system affects the severity of charcoal rot in soybean caused by *M. phaseolina.* A significantly higher percentage of infected roots was detected in plants developed under a conventional system (higher densities of microsclerotia in roots were found), than under no-till system. The most important factor for infection is low soil moisture and because no-till system contributes to less drought stress, it is a better way of preparing soil for soybean production. It is well known that no-till systems are cooler than conventional ones, mainly due to the crop residue layer on the soil surface. Beside low moisture, there is also a high temperature as an important factor for infection [22]. Furthermore, a no-tillage system, with high level of biological diversity, provides good conditions for a plant root development and the avoidance of infection by a soil-borne pathogen [23].

In the cvs. Atlas and Rubin, none of the tested oxidative stress parameters (O_2_^•−^ radical production, SOD activity, and LP process) showed a significant difference compared to their controls. This indicates that the antioxidant system of these two soybean cultivars were not affected with the phytopathogenic fungus *M. phaseolina* or that the infection failed to take place. Furthermore, the lowest lesion length was in the cv. Atlas, followed by the cv. Rubin compared to the cvs. Victoria and Favorit. The results indicate that the cvs. Atlas and Rubin showed the greatest tolerance to *M. phaseolina*. On the other hand, the cv. Victoria is the most sensitive due to the lowest SOD activity, slightly increased O_2_^•−^ radical production, the highest LP level, and the highest length of the lesions. The cultivar Favorit showed high sensitivity to *M. phaseolina* with the highest production of the superoxide anion radical and high length of the lesions. It seems that the oxidative burst and activation of the antioxidant enzymes (SOD) has an important role in the soybean cv. Favorit’s defense against *M. phaseolina*, because it catalyzes the removal of O_2_^•−^ radicals and prevents the formation of a more toxic free radical ^•^OH radical, which causes lipid peroxidation.

The mechanism of soybean tolerance to drought is often equated with resistance to *M. phaseolina*. Therefore, cultivation of genotypes whose reproductive growth stages (from flowering through maturation) does not coincide with the dry period is recommended in the affected areas [24]. Through the long-term monitoring of soybean seed production in Serbia, it was observed that the cv. Rubin (MG ‘II’) was more tolerant compared to the cv. Victoria (MG ‘I’) in drought years. Such observation on early-maturing cultivars did not exist, because their reproductive growth stages do not coincide with dry period and *M. phaseolina* infection. This avoidance of infection led many farmers to believe that early-maturing cultivars were resistant to *M. phaseolina*. However, this research has shown that early-maturing cultivars such as the cv. Atlas (MG ‘0’) can be tolerant, but another can be very susceptible such as the cv. Favorit (MG ‘000’).

## 4. Materials and Methods

**Figure 4:**
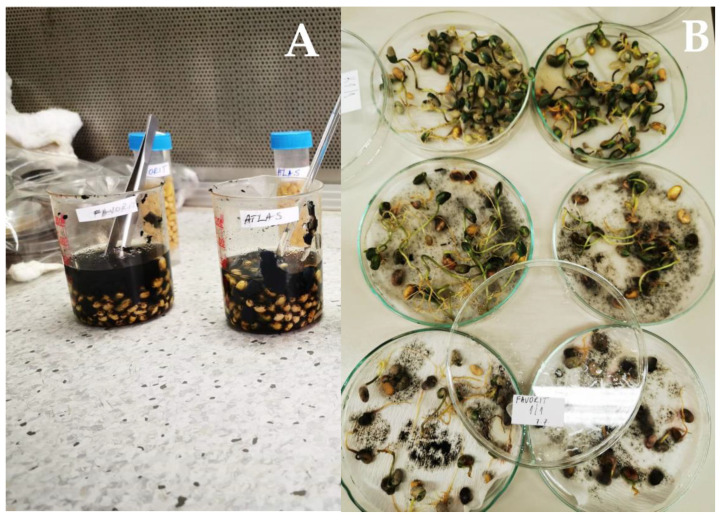
The seed test for the screening of soybean biochemical responses to *M. phaseolina*: (**A**) immersion of seeds in *M. phaseolina* mycelial suspension, and (**B**) inoculated seeds after three days on wet filter paper in 90-mm Petri dishes.

Four soybean cultivars, belonging to different maturity groups (MG), were infected with *M. phaseolina* isolate (MP5 isolate was deposited in the collection of the Institute of Field and Vegetable Crops) that was recovered from infected soybean stem in Rimski Šančevi, Serbia. Since *M. phaseolina* is a soil-inhabiting and seed-borne polyphagous phytopathogenic fungus, the biochemical responses were determined by immersing the seeds of the same soybean cultivars (Favorit MG ‘000’, Atlas MG ‘0’, Victoria MG ‘I’, and Rubin MG ‘II’) for two hours in *M. phaseolina* mycelial suspension [25] (Figure 4A). One hundred soybean seeds of each of the four tested cultivars were randomly chosen using the soybean seed counter (patent registered in Serbia—MP-2020/0007). Twenty-five seeds were placed on wet filter paper in 90-mm Petri dishes (Figure 4B). The experiment was set up in four replications (100 seeds) and incubated at 24 °C in the dark. An equal number of soybean seeds treated with sterile water was used as negative control. After three days of inoculation, the seedlings were sampled to prepare soybean seedling extracts (in triplicates) for further biochemical analyses.

For the determination of the biochemical parameters, 1 g of the fresh plant material (whole seedlings of four soybean cultivars, separately treated with the tested *M. phaseolina* isolate, as well as from the control), were homogenized with 10 mL of the phosphate buffer (KH_2_PO_4_, 0.1 M, pH 7.0). After the centrifugation, the supernatants (soybean seedling extracts) were used for the biochemical analyses, carried out spectrophotometrically, using an UV/VIS spectrophotometer (Thermo Scientific Evolution 220, Waltham, MA, USA).

The amount of produced superoxide anion (O_2_^•−^) was determined by the method of Misra and Fridovich [26]. The total amount of produced superoxide anion radicals was given in nmol O_2_^•−^ per g of fresh weight (nmol O_2_^•−^ g^−1^ FW).

The superoxide dismutase activity (SOD) (EC 1.15.1.1) was assayed according to the method of Mandal et al. [27], which was slightly modified by measuring its ability to inhibit the photochemical reduction of the nitro blue tetrazolium (NBT) chloride. One unit of the SOD activity was defined as the amount of enzymes required to inhibit the reduction of the NBT by 50%. The activity of the enzyme was expressed in unit per g of fresh weight (U g^−1^ FW).

The MDA content, the end product of the cell membrane lipid peroxidation process, was measured at 532 nm using the thiobarbituric acid (TBA) test [27]. The total amount of TBA-reactive substances was given in nmol of MDA equivalents per g of fresh weight (nmol MDA g^−1^ FW).

**Figure 5:**
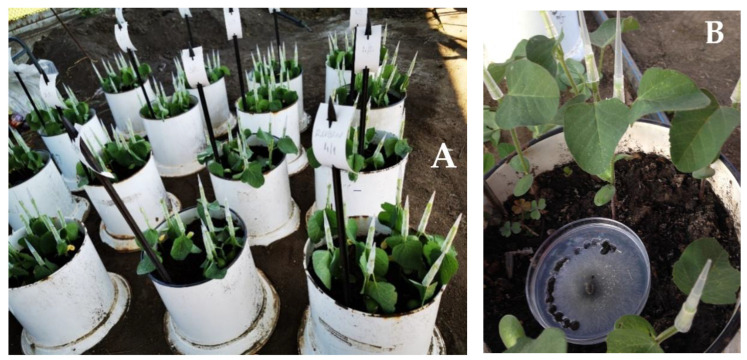
The cut-stem method for screening of soybean tolerance to *M. phaseolina* at V2 growth stage (**A**) and (**B**) circular agar disks with fungal mycelium were taken from the margin of an actively growing *M. phaseolina* culture using a 200-µL pipette tip and placed on the top of the cut stem.

To verify the susceptibility of Favorit, Atlas, Victoria, and Rubin, ten plants of each cultivar were inoculated at V2 growth stage by the cut-stem method [20] which is standardly used for disease resistance screening (Figure 5A). Plants were cut 25 mm above the unifoliolate node, where a circular disk of agar with fungal mycelium was placed. The open end of a 200-µL pipette tip was pushed into the margin of an actively growing *M. phaseolina* culture growing on potato dextrose agar (PDA), and a circular disk of fungal mycelium and agar were cut and removed. The pipette tip containing the agar disk with *M. phaseolina* mycelium was immediately placed over the cut stem and pushed down as far as possible in order to embed the stem and to secure the agar disk on top of the stem (Figure 5B). Control plants were treated with a mycelium-free agar disk. The experiment was repeated three times. Inoculated plants were covered with plastic bags for three days. Afterward, plastic bags and pipette tips were removed, and visual observations of the disease progression and measurement of stem lesions with a ruler were performed every third or fourth day for three weeks.

All measurements were performed in triplicates. Values of the biochemical parameters were expressed as mean ± standard error of mean and tested by ANOVA followed by a comparison of the means by Duncan’s multiple range test (*p* < 0.05). The length of the lesions was expressed by the mean of all measurements and tested by ANOVA followed by the Duncan multiple range test. As a result of the Duncan test, homogenous groups were formed (*p* < 0.05). Data were analyzed using STATISTICA for Windows version 11.0.

## 5. Conclusions

According to the obtained results, it can be concluded that the fungus *M. phaseolina* has different effects on the tested soybean cultivars. The present results indicate that the cvs. Atlas and Rubin have the best performance against *M. phaseolina* in southeast Europe. The most susceptible to *M. phaseolina* was the soybean cv. Victoria with the greatest level of MDA. Furthermore, the cv. Favorit showed high sensitivity to *M. phaseolina* with the highest production of the superoxide anion radical. In conclusion, regarding the potential risk of infection with the fungus *M. phaseolina*, the best soybean cultivar for production is Atlas (MG ‘0’), followed by the cv. Rubin (MG ’II’), because they are tolerant to this pathogen. These results prove the hypothesis that early-maturing cultivars, such as Favorit (MG ‘000’), just avoid the dry period and the moment of *M. phaseolina* infection due to the short growing season. The identification of commercial soybean cultivars tolerant to charcoal rot is important for seed production, especially organic and low input, because tolerance is crucial for minimizing the gap between yield potential and actual yield. Since the microsclerotia of *M. phaseolina* are widely present in the soil, it is essential to grow tolerant soybean cultivars and thus avoid yield losses that can be from 30 to 50%. Furthermore, results from this research will be valuable to soybean breeders in planning future parental selections and in developing cultivars beneficial to soybean producers. This is of utmost importance since there is still no soybean cultivar resistant to *M. phaseolina*.

## Figures and Tables

**Table 1:** Effect of soybean inoculation with *M. phaseolina* on the length of the stem lesions (mm). Different letters indicate statistically significant differences (*p* < 0.05).

Treatment	Length of the Lesions (mm)
Control	0 ^e^
Favorit	98.8 ^b^
Atlas	53 ^d^
Victoria	117.7 ^a^
Rubin	74.9 ^c^

The data above represents the mean values of all the length lesions measurements. ^a–e^ Values without the same superscripts within each column differ significantly (*p* < 0.05).

**Table 2:** Correlation of lesion length with biochemical parameters (O_2_^•−^, SOD and LP). Red marked correlations are significant at *p* < 0.05.

	Length of the Lesions	O_2_^•−^	SOD	LP
Length of the Lesions	1.000000	0.188977	−0.918550	0.458220
O_2_^•−^	0.188977	1.000000	−0.013693	−0.674829
SOD	−0.918550	−0.013693	1.000000	−0.549985
LP	0.458220	−0.674829	−0.549985	1.000000

## Data Availability

Data supporting reported results are linked with the EU Horizon 2020 Project—ECOBREED—Increasing the efficiency and competitiveness of organic crop breeding under Grant No: 771367 and can be found at open repository Zenodo (https://zenodo.org/communities/ecobreed/?page=1&size=20) (accessed on 12 June 2023).

## References

[1] Meseldžija M., Rajković M., Dudić M., Vranešević M., Bezdan A., Jurišić A., Ljevnaić-Mašić B. (2020). Economic feasibility of chemical weed control in soybean production in Serbia. Agronomy.

[2] Gupta G.K., Sharma S.K., Ramteke R. (2012). Biology, epidemiology and management of the pathogenic fungus *Macrophominaphaseolina* (Tassi) Goid with special reference to charcoal rot of soybean (*Glycine max* (L.) Merrill). J. Phytopathol..

[3] Cohen R., Elkabetz M., Paris H.S., Gur A., Dai N., Rabinovitz O., Freeman S. (2022). Occurrence of *Macrophomina phaseolina* in Israel: Challenges for Disease Management and Crop Germplasm Enhancement. Plant Dis..

[4] Wrather J.A., Shannon J.G., Carter T.E., Bond J.P., Rupe J.C., Almeida A.M.R. (2008). Reaction of drought-tolerant soybean genotypes to *Macrophomina phaseolina*. Plant Health Prog..

[5] Abbas H.K., Bellaloui N., Accinelli C., Smith J.R., Shier W.T. (2019). Toxin production in soybean (*Glycine max* L.) plants with charcoal rot disease and by *Macrophomina phaseolina*, the fungus that causes the disease. Toxins.

[6] Kendig S.R., Rupe J.C., Scott H.D. (2000). Effect of irrigation and soil water stress on densities of *Macrophomina phaseolina* in soil and roots of two soybean cultivars. Plant Dis..

[7] Malenčić Đ., Kiprovski B., Stojšin V., Budakov D., Pogančev G. (2014). Comparison of oxidative stress parameters in soybean seedlings inoculated with *Macrophomina phaseolina* from different isolates. Contemp. Agric..

[8] Wang X., Jiang N., Liu J., Liu W., Wang G.L. (2014). The role of effectors and host immunity in plant-necrotrophic fungal interactions. Virulence.

[9] Bellaloui N., Mengistu A., Smith J.R., Abbas H.K., Accinelli C., Shier W.T. (2023). Soybean Seed Sugars: A role in the mechanism of resistance to charcoal rot and potential use as biomarkers in selection. Plants.

[10] Khambhati V.H., Abbas H.K., Sulyok M., Tomaso-Peterson M., Shier W.T. (2020). First report of the production of mycotoxins and Other Secondary Metabolites by *Macrophomina phaseolina* (Tassi) Goid. Isolates from Soybeans (*Glycine max* L.) Symptomatic with charcoal rot disease. J. Fungi.

[11] Khambhati V.H., Abbas H.K., Sulyok M., Tomaso-Peterson M., Chen J., Shier W.T. (2023). Mellein: Production in culture by *Macrophomina phaseolina* isolates from soybean plants exhibiting symptoms of charcoal rot and its role in pathology. Front. Plant Sci..

[12] Malenčić Đ., Kiprovski B., Popović M., Prvulović D., Miladinović J., Đorđević V. (2010). Changes in antioxidant systems in soybean as affected by *Sclerotinia sclerotiorum* (Lib.) de Bary. J. Plant Physiol. Biochem..

[13] Malenčić Đ., Popović M., Miladinović J. (2003). Stress tolerance parameters in different genotypes of soybean. Biol. Plant..

[14] Begum N., Hasanuzzaman M., Li Y., Akhtar K., Zhang C., Zhao T. (2022). Seed Germination Behaviour, Grouth, Physiology and Antioxidant Metabolism of Four Contrasting Cultivars under Combined Drought and Salinity in Soybean. Antioxidants.

[15] Rani A., Kiran A., Sharma K.D., Prasad P.V.V., Jha U.C., Siddique K.H.M., Nayyar H. (2021). Cold Tolerance during the Reproductive Phase in Chickpea (*Cicer arietinum L.*) Is Associated with Superior Cold Acclimation Ability Involving Antioxidants and Cryoprotective Solutes in Anthers and Ovules. Antioxidants.

[16] Helepciuc F.E., Mitoi M.E., Aldea F., Matei S., Matei G.M., Cogălniceanu G. (2014). Antioxidant response in soybean cell suspensions treated with fungal elicitors. Olten. Stud. Ştiinţele Nat..

[17] Dey P., Biswas C., Karmakar P.G. (2016). Identification and characterization of differentially expressed novel miRNAs (21–24 nt) in a *Macrophominaphaseolina* resistant RIL line of jute (*Corchorus capsularis* L.). Physiol. Mol. Plant Pathol..

[18] Marquez N., Giachero M.L., Declerck S., Ducasse D.A. (2021). *Macrophominaphaseolina*: General Characteristics of Pathogenicity and Methods of Control. Front. Plant Sci..

[19] Wang G., Wang P., Lin Y., Zhang L.B., Wu Y. (2002). The studies of sensitivity of cultivars in soybean to lines of *Agrobacterium tumefaciens*. Yi Chuan.

[20] Twizeyimana M., Hill C.B., Pawlowski M., Paul C., Hartman G.L. (2012). A Cut-Stem Inoculation Technique to Evaluate Soybean for Resistance to *Macrophominaphaseolina*. Plant Dis..

[21] Al-Ahmadi A.H., Subedi A., Wang G., Choudhary R., Fakhoury A., Watson D.G. (2018). Detection of charcoal rot (*Macrophomina phaseolina*) toxin effects in soybean (*Glycine max*) seedlings using hyperspectral spectroscopy. Comput. Electron. Agric..

[22] Almeida A.M.R., Amorim L., Filho A.B., Torres E., Farias J.R.B., Benato L.C., Pinto M.C., Valentim N. (2003). Progress on soybean charcoal rot under tillage and no-tillage systems in Brazil. Fitopatol. Bras..

[23] Perez-Brandán C., Arzeno J.L., Huidobro J., Grümberg B., Conforto C., Hilton S., Bending G.D., Meriles J.M., Vargas-Gil S. (2012). Long-term effect of tillage systems on soil microbiological, chemical and physical parameters and the incidence of charcoal rot by *Macrophomina phaseolina* (Tassi) Goid in soybean. J. Crop. Prot..

[24] Mengistu A., Arelli P.A., Bond J.P., Shannon G.J., Wrather A.J., Rupe J.B., Chen P., Little C.R., Canaday C.H., Newman M.A. (2011). Evaluation of Soybean Genotypes for Resistance to Charcoal Rot. Plant Health Prog..

[25] Petrović K. (2012). Morphological, Molecular and Pathogenic Characterization of Species *Diaporthe/Phomopsis* on Soybean in Serbia. Ph.D. Dissertation.

[26] Misra H.P., Fridovich I. (1972). The role of superoxide anion in the autoxidation of epinephrine and a simple assay for superoxide dismutase. J. Biol. Chem..

[27] Mandal S., Mitra A., Mallick N. (2008). Biochemical characterization of oxidative burst during interaction between *Solanum lycopersicum* and *Fusarium oxysporum* f. sp.. lycopersici. Physiol. Mol. Plant Pathol..

